# Global Assessment of Marine Reptiles and Mammals Using a Taxonomic Distinctness Tool: Implications for Their Conservation

**DOI:** 10.1002/ece3.72537

**Published:** 2025-11-23

**Authors:** Laura Fuentes‐Tejada, Davinia Torreblanca, José Carlos Báez

**Affiliations:** ^1^ Department of Biodiveristy, Ecology, and Evolution Complutense University of Madrid Madrid Spain; ^2^ Facultat de Ciències de la Terra Universitat de Barcelona (UB) Barcelona Spain; ^3^ Instituto Español de Oceanografía (IEO, CSIC) Fuengirola Spain; ^4^ Instituto Iberoamericano de Desarrollo Sostenible (IIDS) Universidad Autónoma de Chile Temuco Chile

**Keywords:** biogeography, evolution, mammals, marine protected areas, phylogeography, reptiles

## Abstract

Understanding the spatial distribution of marine biodiversity is essential for assessing evolutionary processes and informing conservation priorities. This study investigates global distribution patterns of marine reptiles and mammals—two clades with contrasting evolutionary histories. Marine reptiles, representing lineages with arrested diversification, have undergone extensive extinction events since the Mesozoic, while marine mammals are characterized by recent and rapid diversification, particularly during the Miocene. Using taxonomic distinctness (Δ^+^) and species richness across 15 oceanic regions, we evaluated whether observed distribution patterns deviate from null expectations derived from randomized assemblages. Our results reveal that marine mammals exhibit significantly non‐random, patterned distributions, with higher‐than‐expected taxonomic distinctness in the Arctic and North Pacific, indicating evolutionary radiation and lineage diversification in these regions. Conversely, marine reptiles generally conform to random expectations, with lower‐than‐expected Δ^+^ values in the Tropical Pacific and Indian Oceans—areas dominated by closely related sea snakes—suggesting phylogenetic clustering and historical lineage loss. These results support our initial hypothesis that diversification dynamics shape current species distributions. Specifically, rapidly diversifying groups lead to phylogenetic clustering (i.e., creating local assemblages of closely related species), while lineages with arrested diversification show overdispersed or clustered patterns. Importantly, we identify key conservation hotspots, highlighting the Indo‐Pacific for marine reptiles and the Arctic–North Pacific for marine mammals. These findings underscore the necessity of integrating taxonomic distinctness and species richness to guide the strategic expansion of Marine Protected Areas (MPAs). As ocean warming and anthropogenic pressures continue to reshape the distribution of marine species, understanding macroecological and phylogeographic patterns becomes vital for ensuring the efficient protection of marine biodiversity.

## Graphical abstract

This study examines the global distribution of marine reptiles and mammals, revealing contrasting biogeographic patterns shaped by their distinct evolutionary histories. Marine mammals exhibit non‐random distributions with elevated taxonomic distinctness in the Arctic and North Pacific, whereas marine reptiles show phylogenetic clustering in tropical regions, reflecting historical lineage loss. These findings emphasize the importance of integrating species richness and taxonomic distinctness to inform the strategic expansion of Marine Protected Areas and enhance conservation planning.

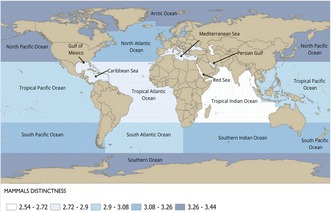

## 1 Introduction

Rapidly diversifying lineages display different phylogenetic species distributions than lineages with arrested diversification (Sanmartín 2012; Caron and Pie 2022). Specifically, studies have shown that at a macroevolutionary spatial and temporal scale, stable and established lineages exhibit non‐random or patterned distributions while lineages that have undergone rapid local radiation events exhibit overdispersed distributions (Caron and Pie 2022). Thus, studying lineage distributions can offer valuable insight into species' past, present, and future status, and consequently their conservation palaeobiology. Marine reptiles and mammals present a unique opportunity for assessing the status and trends of lineages as these groups have evolved from terrestrial counterparts from specific dispersal centers, and exhibit distinct lineage trajectories (i.e., rapid or declining species diversification; Motani 2009; Berta et al. 2015). Marine reptiles are an example of arrested diversification (i.e., a phenomenon in which lineages exhibit lower rates of diversification; Caron and Pie 2022), with this paraphyletic group (i.e., sharing a common ancestor but not including all its descendants; Sturmbauer 2013) experiencing a long‐term macroevolutionary bottleneck since their diversity peak in the Mesozoic (~252 to 66 million years ago; Motani 2009; Thorne et al. 2011). Marine reptiles faced two major extinction events during the late Triassic and the Cretaceous‐Tertiary boundary, causing significant species losses (Bardet 1994). Indeed, there are currently 91 living species of marine reptiles, including nine turtles, two crocodiles, 79 snakes, and one iguana (Báez et al. 2024). In contrast, marine mammal lineages have experienced rapid diversification since they transitioned from a terrestrial to an aquatic environment, likely around 50 million years ago (Schenk et al. 2013; Steinthorsdottir et al. 2021). Today, marine mammals comprise 128 species of marine mammals belonging to 18 families. There are an additional two species that recently became extinct due to anthropogenic factors: the sea mink *Neovison macrodon* , and Steller's sea cow *Hydrodamalis gigas* . Moreover, the evolutionary history of lineages has led to their distinct geographic distributions. For example, there are no sea snakes in the Atlantic, or sirenians in the North Pacific and the Mediterranean Sea. Simultaneously, the marine iguana can only be found in the Galapagos Islands of the Pacific Ocean.

Multiple biogeographical events have influenced the present‐day distributions of marine species (Sanmartín and Ronquist 2004; Bowen et al. 2013). One example is the Messinian Salinity Crisis ~5.6 million years ago, which caused the nearly complete desiccation of the Mediterranean Sea and its subsequent inundation originating from the Atlantic Ocean through the Strait of Gibraltar (Hsü et al. 1977; Krijgsman et al. 1999; Agiadi et al. 2024). Through shifting environmental conditions, the Messinian Salinity Crisis significantly impacted the current marine biodiversity in the Mediterranean (Krijgsman et al. 1999; Costa et al. 2022). Additionally, the formation of oceanic and terrestrial barriers, such as the separation of the Atlantic and Pacific oceans by the Isthmus of Panama ~3 million years ago, caused the divergence and speciation of species in different oceanic basins (Lessios 2008). Similarly, changes in oceanic currents and sea temperatures over geologic time have influenced the capacity for dispersal and survival of species in different regions (Renema et al. 2008).

In addition to providing historical information, current species distributions can give insights into how species might react to future climatic events. In recent years, several studies have documented changes in the environment driven by climate change, such as annual increases in oceanic temperatures, as well as changes in oceanic currents and acidification levels (Garcia‐Soto et al. 2021; Cheng et al. 2022; Hastings et al. 2020; Guinotte and Fabry 2008). These climate change‐induced environmental shifts will affect marine species' future distributions and abundance (Perry et al. 2005; Hastings et al. 2020; Bryndum‐Buchholz et al. 2019). Due to subsequent reductions in nutrient and habitat availability, species are likely to become more vulnerable to threats (Poloczanska et al. 2016).

Multiple studies have questioned the efficiency of traditional diversity measures (e.g., Shannon and Simpson diversity indices; von Euler and Svensson 2001; Clarke and Warwick 1998). Both the Shannon and Simpson indices have several disadvantages since they are influenced by sample size and effort (Morris et al. 2014). Clarke and Warwick (1998) proposed the use of taxonomic distinction (TD) for presence/absence data. One of the main benefits of using this method is its independence from sampling effort and area of study, which is useful when using historical data and enables the analysis of regions of different sizes, such as ocean basins and seas (Clarke and Warwick 1998; Salvo Tierra et al. 2021). This index measures diversity through evolutionary similarity between species, using the taxonomic distance between any two randomly chosen species in a sample (Tolimieri and Anderson 2010). It is important to distinguish between taxonomic diversity (TD) and species richness, that is, the number of species present in a given area. While TD is useful for assessing the taxonomic structure of a group, species richness is valuable for understanding the abundance of these species in a specific area (Ellingsen et al. 2005). Thus, Taxonomic Distinctness and species richness can be used for identifying areas for the conservation of lineages or for the conservation of biodiversity, respectively.

In this study, we assess global distribution patterns of marine reptiles and mammals: two important components of marine biodiversity that currently face multiple threats to their survival. We use taxonomic distinction and species richness to assess large‐scale distribution patterns of these two groups and the subgroups that make them up (i.e., turtles, sea snakes, cetaceans, and pinnipeds). Because we are aiming to delineate important areas for the protection of lineages and not only biodiversity or specific habitats, we included all species that forage, inhabit, or have been incidentally caught by fisheries in the open ocean. We predict that each group and subgroup will have distinct distributional patterns. For example, some groups will exhibit patterned or non‐random distributions, likely indicating specific phylogeographic distributions, while others will exhibit random or “mosaic” distributions.

## 2 Materials and Methods

### 2.1 Information Sources

A list of marine reptiles was obtained from Báez et al. (2024). A list of marine mammals, including recently extinct species, was obtained from the Society for Marine Mammalogy (Committee on Taxonomy 2024). Using Excel, columns for species, genus, family, order, and class were made. Presence (marked by a 1) and absence (marked by a 0) of each species were listed for each ocean/sea: North Atlantic, Tropical Atlantic, South Atlantic, North Pacific, Tropical Pacific, South Pacific, Tropical Indian, Southern Indian, Arctic, Southern, Mediterranean Sea, Red Sea, Persian Gulf, Gulf of Mexico, and the Caribbean Sea. We determined each zone based on the following coordinates: Arctic +60 and above, North 60°–23°, Tropical 23° to −23°, South −23° to −60°, Southern −60 and below. These subdivisions were chosen due to the distinct environmental conditions at each of these latitudes, which could affect species distributions (Rabosky et al. 2018).

The individual geographical distributions of each species were obtained through the information available on the IUCN website, using the information available in the evaluation of each species (IUCN 2025, https://www.iucnredlist.org/). In the cases where the species were not included in the IUCN, their distribution information was obtained through GBIF and research papers that described the geographical locations where the species had been observed. Google Scholar was used as the search engine, and we used the following keywords: “(scientific name of the species)” + “geographical distribution”. We only considered presence if there were more than two observations in a region. An observation was counted if the species was present anywhere in the latitudinal and longitudinal range of each region.

### 2.2 Analysis

The TD index (∆+) is calculated using the following formula:

$$∆+=\left[\;\Sigma \sum_{i<j}w_{\mathrm{ij}}\right]/\left[\frac{s\left(s-1\right)}{2}\right]$$

where *s* represents the number of species in the sample and *w*_*ij*_ represents the “distinctness weight” of the length between species *i* and species *j* in the taxonomy. For example, *w*_*ij*_ *= 0* if species *i* and *j* are in the same genus, and *w*_*ij*_ *= 1* if the species belong to the same family. Taxonomic distinctness including 95% confidence intervals (i.e., upper and lower limits) was calculated from 10,000 random replicates from the pooled dataset (all samples/species). TDs were calculated using PAST software (PAST v4.17, 2024).

To perform direct statistical comparisons using a hypothesis testing framework, we generated a null distribution of Δ^+^ using random species combinations. This comparison is grounded in the principle that Taxonomic Distinctness represents an average measure of the phylogenetic diversity within a region, which is influenced by multiple factors such as the geographic distribution of species, paleobiogeographic origin, among others. In other words, it is not solely determined by random chance.

To test the hypothesis, we created 3 null models for each group tested (i.e., marine reptiles, marine mammals, turtles, snakes, cetaceans, and pinnipeds). Each null model had the same number of species in each region as the original dataset, but the species were randomized. For example, if there are seven species of marine reptiles in the North Atlantic Ocean, the null model also included seven species of marine reptiles in the North Atlantic, but the species were different from those in the original dataset. This process yields a null distribution of Δ^+^ based on random species combinations.

Subsequently, two sets of Taxonomic Distinctness values were obtained: one derived from the actual distribution Dataset A, and another 3 from the randomized distributions Dataset B. From these, the mean Taxonomic Distinctness for each set was calculated. A Mann–Whitney *U* test was then employed to determine whether significant differences exist between the means. This procedure was repeated on three separate occasions.

Our hypothesis posits that, if significant differences are detected, it would suggest the existence of a phylogenetic pattern underlying the current distribution—one shaped by evolutionary processes, such as regional radiation events that have driven the present‐day species distribution. Conversely, the absence of significant differences between the observed and randomized patterns would indicate the presence of additional processes that have obscured any historical distributional patterns (e.g., extinction events or localized radiation events). All analyses were performed using PAST4 (Hammer et al. 2001) and R v4.3.2.

## 3 Results

### 3.1 Taxonomic Distinctness for Marine Mammals

**FIGURE 1:**
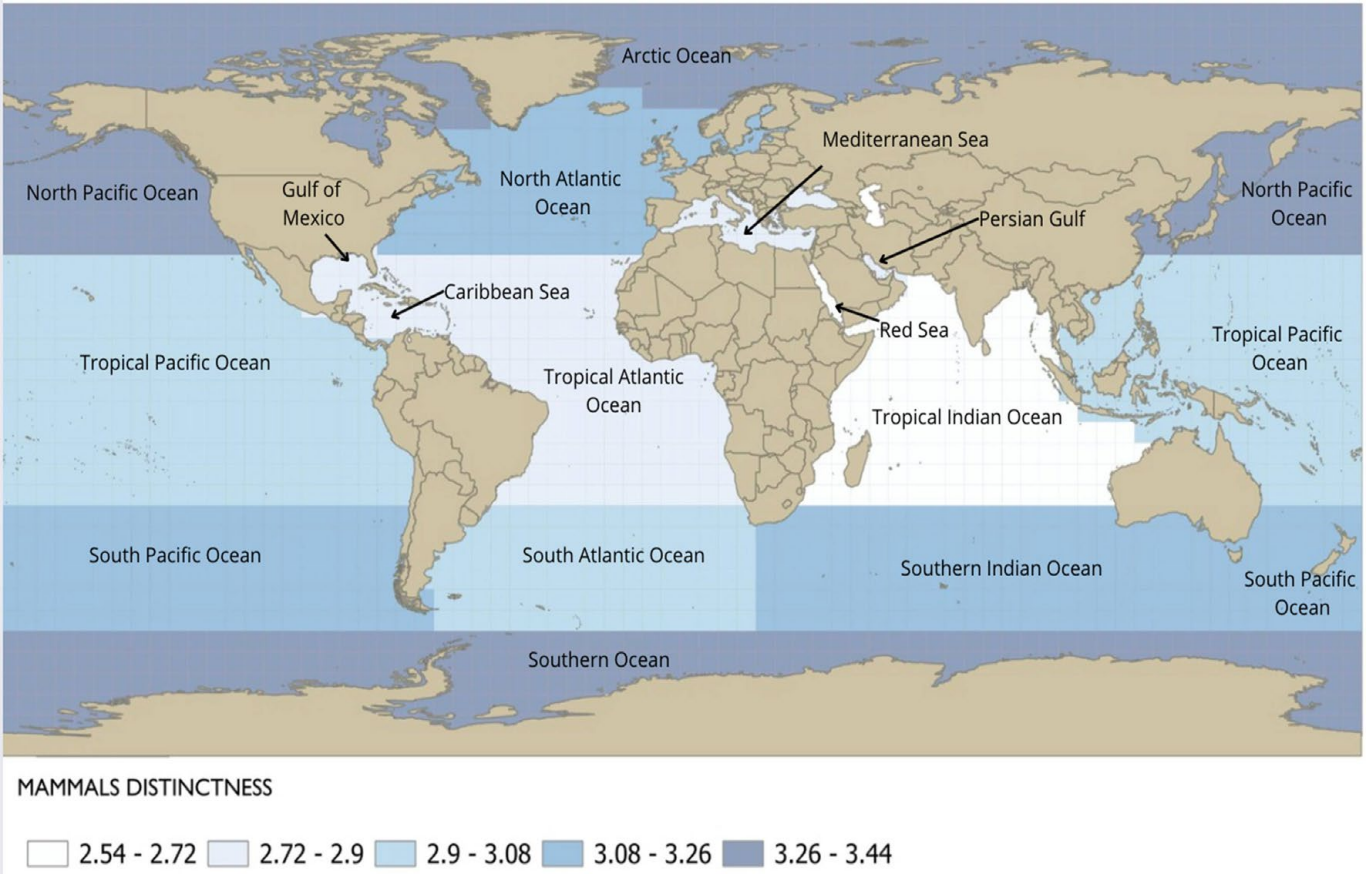
Map showing the delineated regions and their respective taxonomic distinctness values for marine mammal species in each region.

**FIGURE 2:**
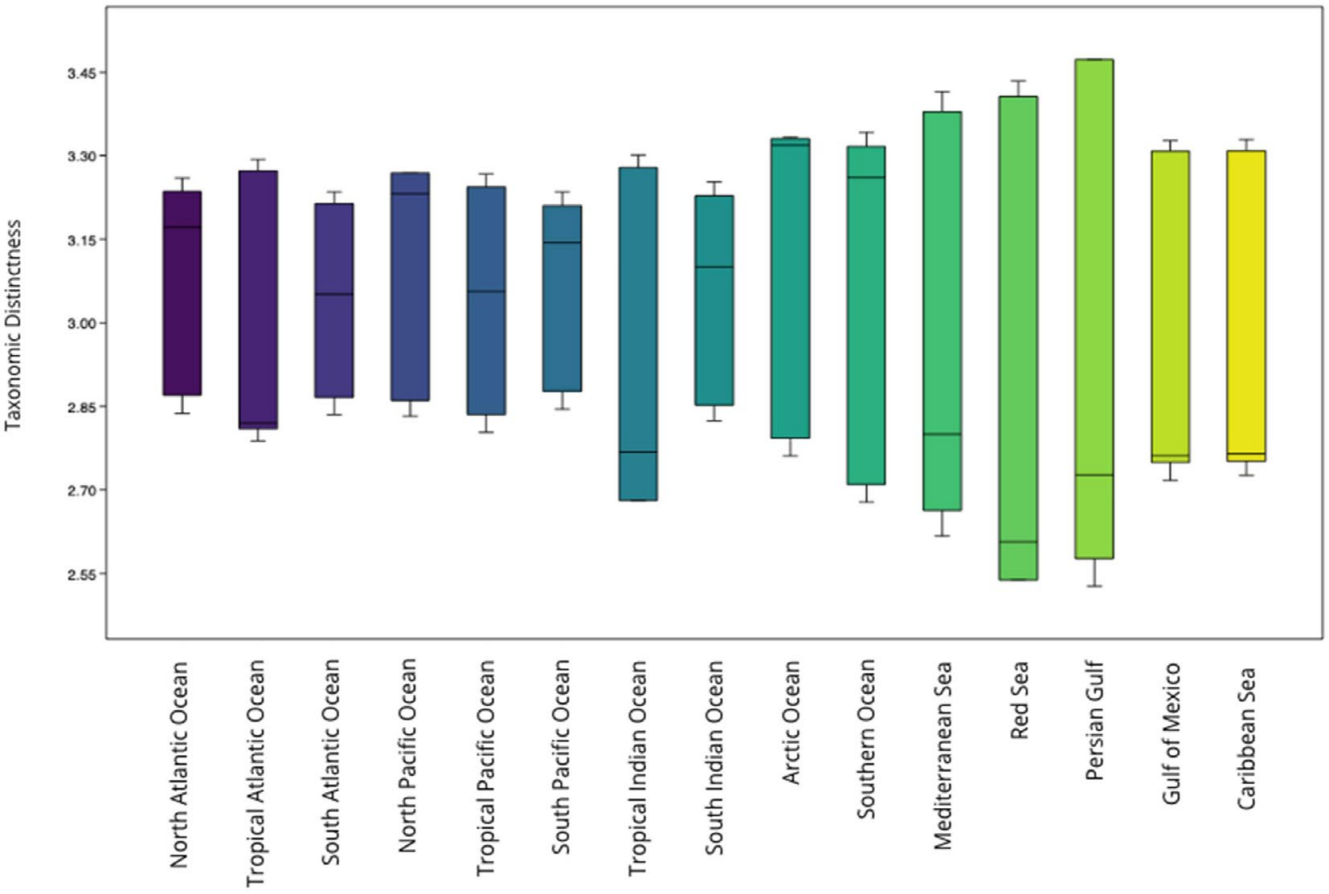
Box plot and results showing ∆+ values with 95% confidence interval ranges (difference between upper and lower limits) for marine mammals in each region.

Figure 1 shows the delineated regions and their respective taxonomic distinctness values for marine mammal species. Marine mammals showed higher than expected diversity in the Arctic (Δ^+^ = 3.33; CI: 2.78–3.33) and the North Pacific (Δ^+^ = 3.269; CI: 2.88–3.25) as seen by the distinctness values being greater than the upper 95% confidence interval (CI), indicating a higher level of taxonomic distinctness in these regions (Figure 2). In contrast, the Red Sea (Δ^+^ = 2.538; CI: 2.623–3.436), the Tropical Indian Ocean (Δ^+^ = 2.681; CI: 2.823–3.298), and the Gulf of Mexico (Δ^+^ = 2.76; CI: 2.775–3.340) showed lower taxonomic distinctness than the expected values with observed values below the lower 95% confidence limit (Figure 2), indicating a higher degree of taxonomic similarity among species of these regions.

We observed a significant difference between marine mammals' observed taxonomic distinction values and those expected by chance from the Mann–Whitney *U* test (U‐statistic = 88.5, *p* < 0.05, *z* = 4.2425). Monte Carlo permutation test confirmed this result (*p* < 0.05), indicating a statistically significant difference in medians between the original and null datasets.

**FIGURE 3:**
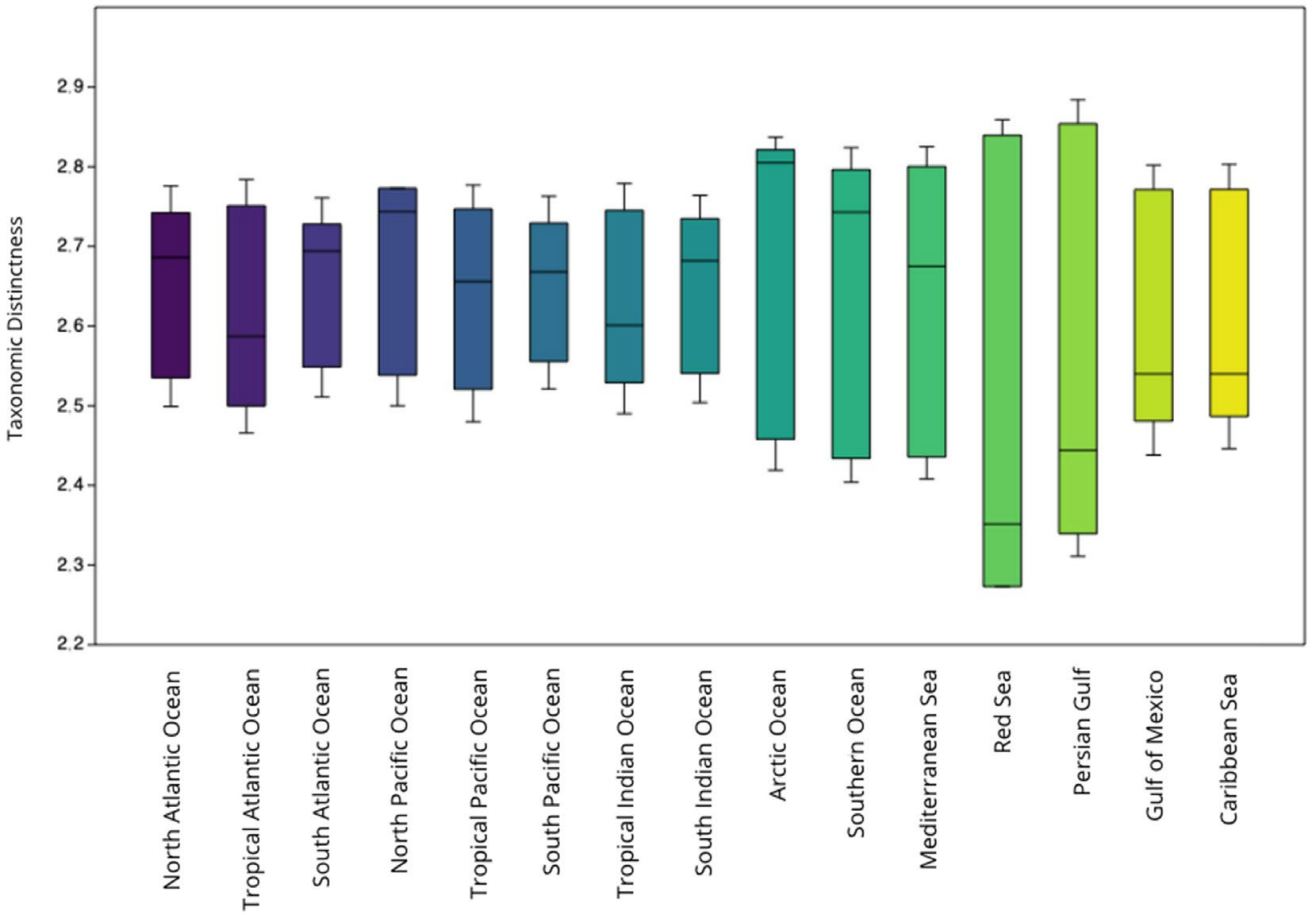
Box plot and results showing ∆+ values with 95% confidence interval ranges (difference between upper and lower limits) for cetaceans in each region.

Cetaceans exhibited higher than expected diversity in the North Pacific (Δ^+^ = 2.773; CI: 2.5–2.722) and lower than expected diversity in the Red Sea (Δ^+^ = 2.273; CI: 2.318–2.833) (Figure 3). We did not observe a significant difference between cetaceans' observed taxonomic distinction values and those expected by chance from the Mann–Whitney *U* test (U‐statistic = 244, *p* > 0.05, *z* = 1.5879). This result was confirmed by the Monte Carlo permutation test (*p* > 0.05).

**FIGURE 4:**
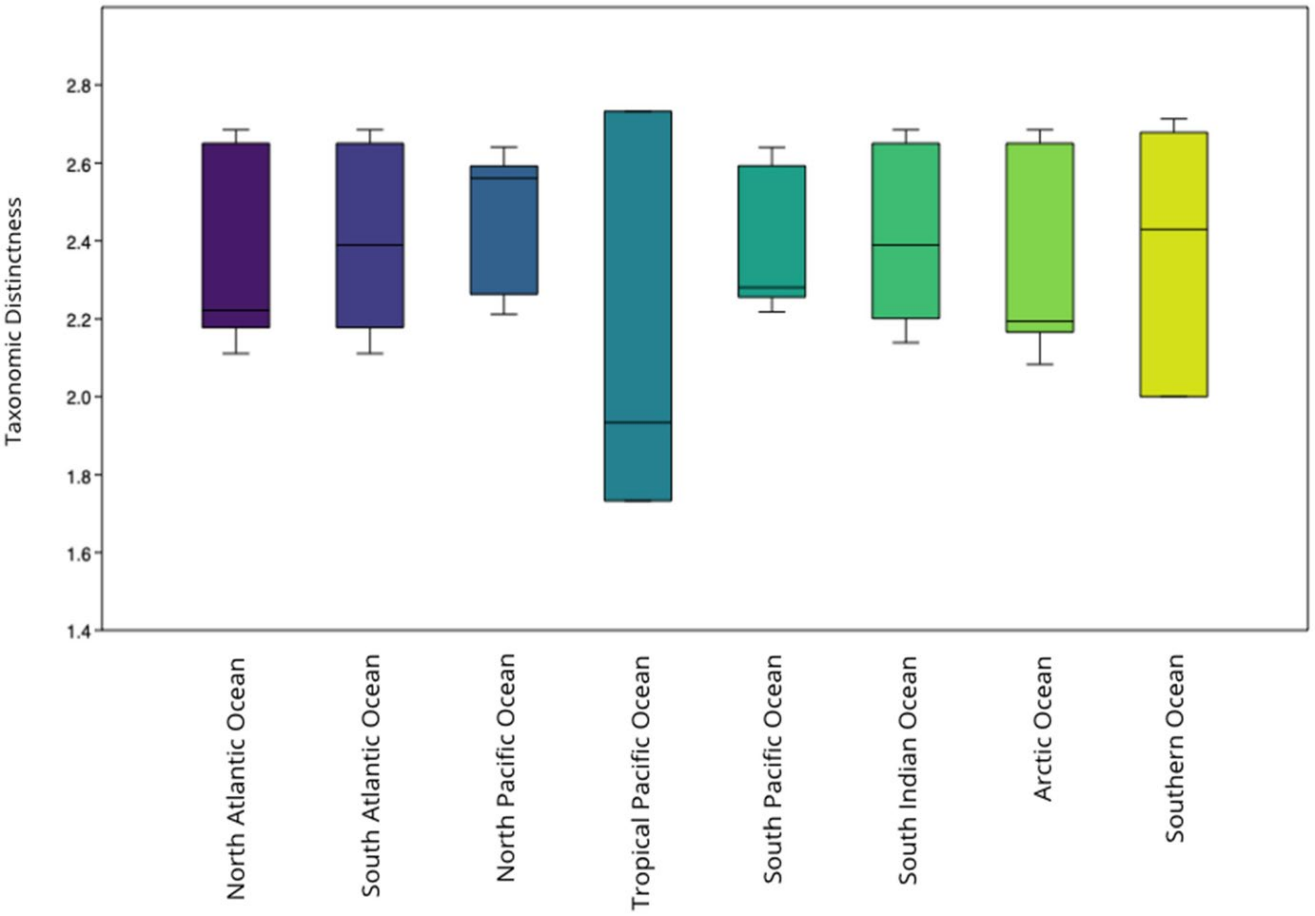
Box plot and results showing ∆+ values with 95% confidence interval ranges (difference between upper and lower limits) for pinnipeds in each region.

Pinnipeds exhibited higher than expected diversity in the Tropical Pacific Ocean (Δ^+^ = 1.733; CI: 1.867–2.733) and lower than expected TD in the Southern Ocean (Δ^+^ = 2.429; CI: 2–2.667) (Figure 4). We observed a significant difference between pinnipeds' observed TD and those expected by chance (U‐statistic = 28, *p* < 0.05, *z* = 2.9427), confirmed by Monte Carlo permutation test (*p* < 0.05).

### 3.2 Taxonomic Distinctness for Marine Reptiles

**FIGURE 5:**
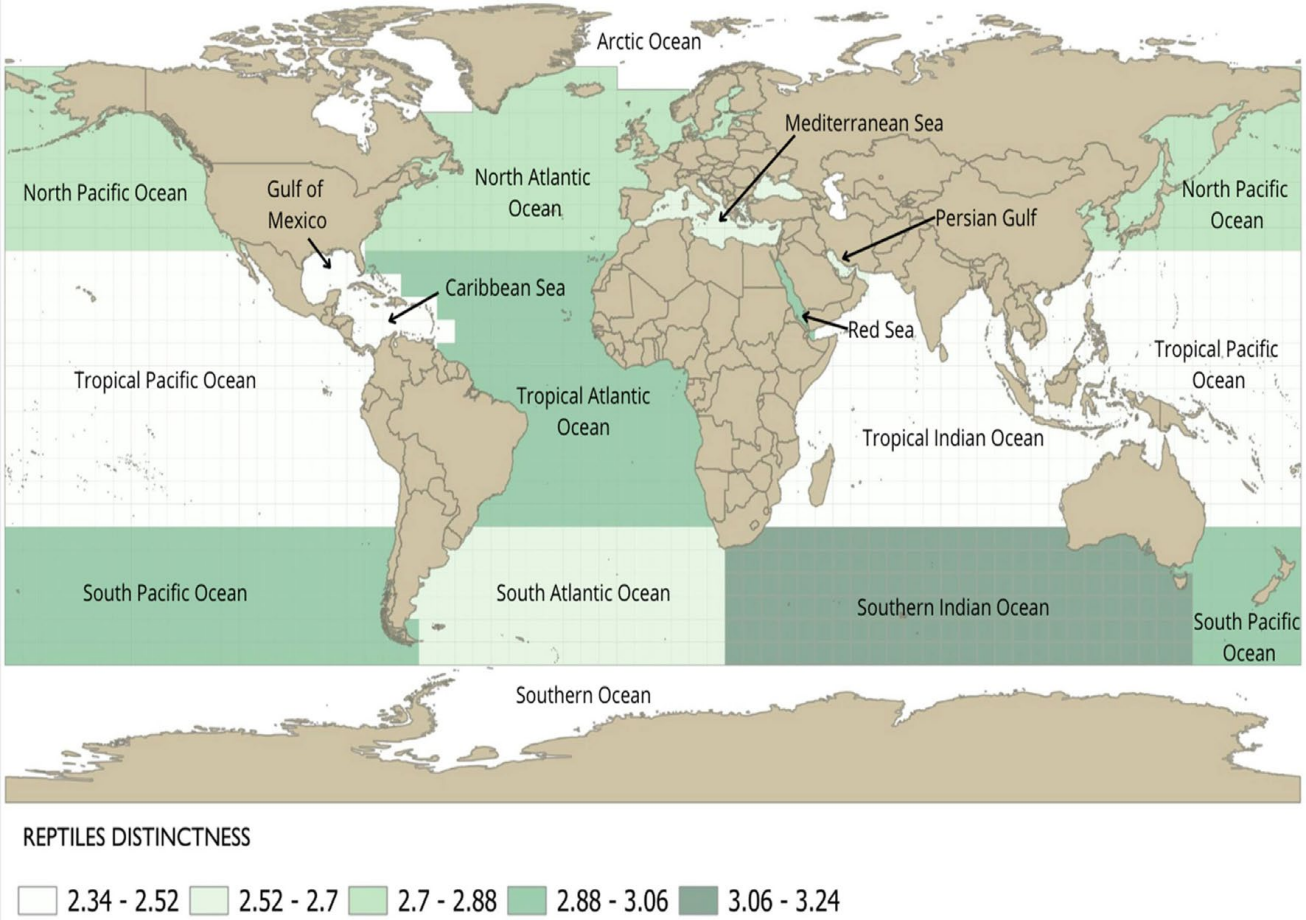
Map showing the delineated regions and their respective taxonomic distinctness values for marine reptile species in each region.

**FIGURE 6:**
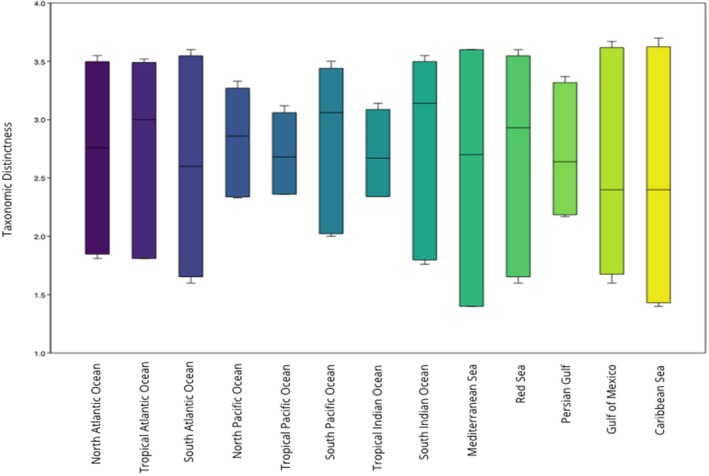
Box plot and results showing ∆+ values with 95% confidence interval ranges (difference between upper and lower limits) for marine reptiles in each region.

Figure 5 shows the delineated regions and their respective taxonomic distinctness values for marine reptile species. For marine reptiles, most regions showed Δ^+^ values that fell within the CI (Figure 6), indicating no significant difference between the observed values and random expectations. However, two regions exhibited lower taxonomic distinctness than expected: the Tropical Pacific (Δ^+^ = 2.36; CI: 2.70–3.12) and the Tropical Indian Ocean (Δ^+^ = 2.34; CI: 2.69–3.14). These results indicate that these regions are composed of closely related taxa. Marine reptiles showed no Δ^+^ values above the upper CI (Figure 6).

We did not observe a significant difference between the observed and random taxonomic distinction of marine reptiles from the Mann–Whitney *U* test (U‐statistic = 176, *p* = 0.104, *z* = 1.6276). Monte Carlo permutation test confirmed this result (*p* > 0.05), indicating no statistically significant difference in means between the original and null datasets.

**FIGURE 7:**
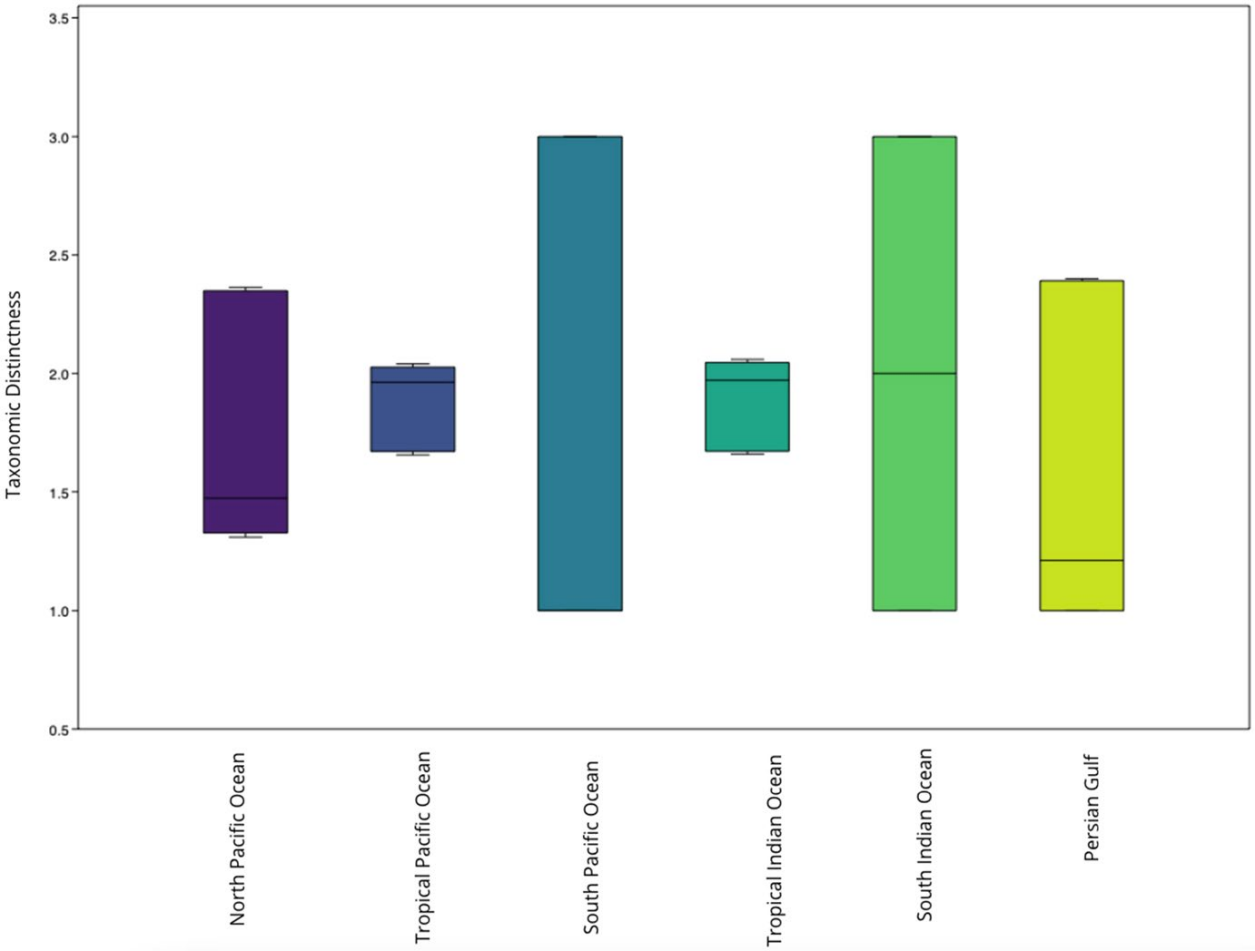
Box plot and results showing ∆+ values with 95% confidence interval ranges (difference between upper and lower limits) for marine snakes in each region.

Marine snakes exhibited the highest TD in the Southern Indian Ocean (Δ^+^ = 2; CI: 1–3) (Figure 7). We did not observe a significant difference between marine snakes' observed TD values and those expected by chance (U‐statistic = 80.5, *p* > 0.05).

**FIGURE 8:**
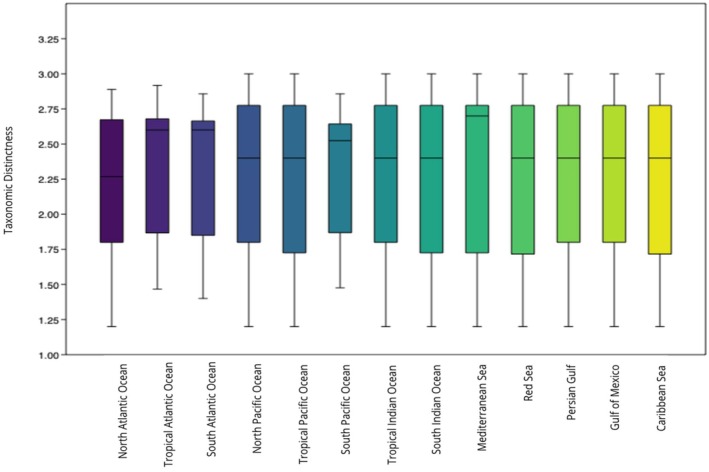
Box plot and results showing ∆+ values with 95% confidence interval ranges (difference between upper and lower limits) for marine turtles in each region.

Marine turtles had Δ^+^ values that fell within the CI and had the highest TD in the Mediterranean Sea (Δ^+^ = 2.7; CI: 1.2–2.7) (Figure 8). We observed significant differences between marine turtles' TD values and those expected by chance (U‐statistic = 160.5, *p* < 0.05, *z* = 1.9766).

### 3.3 Species Richness

**FIGURE 9:**
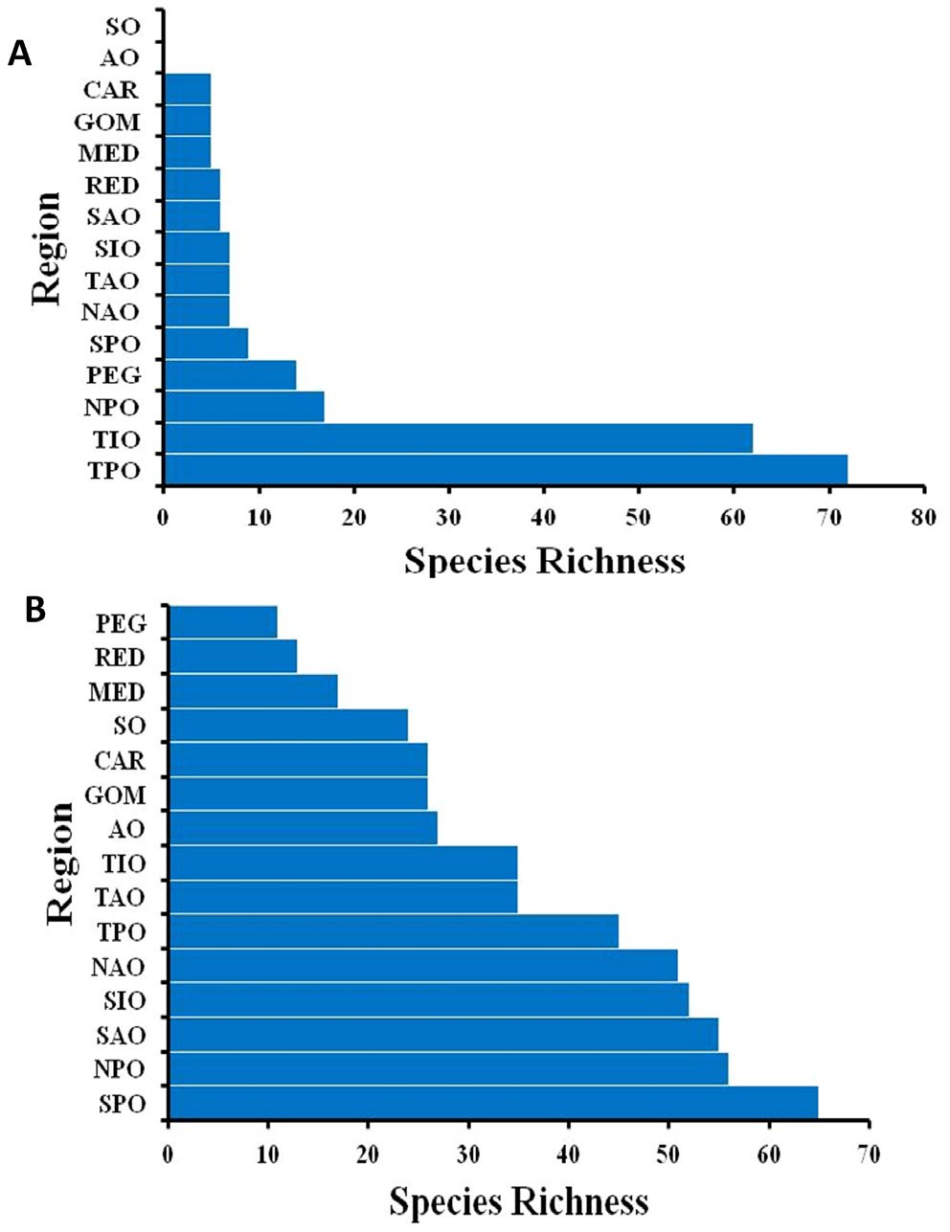
Species richness of marine reptiles (a) and mammals (b) from highest (top) to lowest (bottom) in each region. The regions are the North Atlantic Ocean (NAO), Tropical Atlantic Ocean (TAO), South Atlantic Ocean (SAO), North Pacific Ocean (NPO), Tropical Pacific Ocean (TPO), South Pacific Ocean (SPO), Tropical Indian Ocean (TIO), South Indian Ocean (SIO), Arctic Ocean (AO), Southern Ocean (SO), Mediterranean Sea (MED), Red Sea (RED), Persian Gulf (PEG), Gulf of Mexico (GOM), and the Caribbean (CAR).

We observed the highest number of species in the South Pacific for marine mammals, while marine reptiles had the highest species richness in the Tropical Pacific. The lowest richness for marine mammals was in the Persian Gulf, the Red Sea, and the Mediterranean, while marine reptiles had the lowest richness in the Caribbean, Gulf of Mexico, and the Mediterranean (Figure 9).

## 4 Discussion

We observed that groups that have undergone rapid and local radiation events exhibit a random distribution (i.e., the difference between the observed and random TD was not significant) while groups that have an ancient evolutionary trajectory exhibit patterned or distinct distributions (i.e., the differences between the observed and random TD were statistically significant). For example, we observed a random distribution in sea snakes and cetaceans, while we found a patterned distribution in pinnipeds and turtles. Our findings define regions of high taxonomic distinctness for each lineage, raising questions about whether conservation plans could focus on protecting marine lineages rather than focusing conservation efforts on biodiversity. These findings could guide conservation plans as current Marine Protected Areas (MPAs) do not protect the evolutionary history of species (Mouillot et al. 2016).

### 4.1 Taxonomic Distinctness Comparison Between Groups

Marine mammals exhibited higher TD than expected under a null model in the Arctic and North Pacific regions. Previous studies have identified the North Pacific as an important zone for marine mammal conservation, emphasizing its high species richness for this group (Pompa et al. 2011). However, these same studies have not supported any conservation zones for marine mammals in the Arctic, likely due to low species richness in the region. This demonstrates that taxonomic distinction gives us different information than species richness which can still be valuable for the efficient conservation of lineages and taxonomic diversity. Regions of lower TD than expected under the null model for marine mammals were the Tropical Indian Ocean, the Gulf of Mexico, and the Red Sea, indicating that marine mammal species found in these areas are more closely related taxonomically. The regions of high TD represent adaptive radiation events of marine mammals in northern latitudes (e.g., the significant diversification of marine mammals during the Miocene epoch). Within marine mammals, cetaceans and pinnipeds exhibited higher TD in the North Pacific region, indicating an important zone for the conservation of cetaceans. Notably, Pompa et al. (2011) found the highest concentration of marine mammals that are near extinction in the North Pacific Ocean.

The regions of higher taxonomic diversity could be due to the relative isolation of the Arctic, which could have facilitated the evolution of lineages adapted to the extreme habitats. An example is the polar bear *Ursus maritimus*, which evolved in the Arctic during the Pleistocene and has not been observed in other oceans due to its dependence on sea ice for hunting and survival (Harington 2008). In contrast, the cetaceans and pinnipeds adapted to the polar waters started moving south during the ice ages of the Pleistocene (Harington 2008). Another explanation for the high TD in the Arctic is its low species richness, which increases the probability of the two randomly chosen species being taxonomically distant. These results show a need for consideration of the Arctic region as an important area to protect the taxonomic diversity of marine mammals.

We observed differences between the distributional patterns of each group and subgroup. Specifically, we found that cetaceans did not have patterned phygeographic distributions according to their TD values. This could be due to the relative modernity of the group and subsequent rapid diversification (Arnason et al. 2004; Chen et al. 2011). In contrast, pinnipeds exhibited patterned distributions, which could be due to the group having experienced constant diversification rates (Park et al. 2024).

Marine reptiles exhibited no higher TD compared to expected values. However, they did show lower than expected TD values in the Tropical Indian and Tropical Pacific Oceans, likely due to the phylogenetic clustering of sea snakes in these regions. The high number of sea snakes in these regions increases the probability of randomly choosing two taxonomically closely related species due to their relatedness with most belonging to the family Elapidae (67 snakes out of 79; Báez et al. 2024; Sikdar et al. 2025). Within marine reptiles, we observed that marine turtles exhibited patterned distributions. This is possibly due to the group being relatively ancient, allowing sufficient time for establishing phylogeographic patterns. Sea snakes, however, did not show patterned distributions, likely due to the rapid localized diversification of the group (Kishida et al. 2025). Indeed, marine reptiles showed random distributions when all groups were included, but if marine snakes were excluded, marine reptiles exhibited patterned distributions.

### 4.2 Comparing Species Richness Across Regions

Marine mammals had the highest species richness in the South and North Pacific regions (Figure 6), while for marine reptiles, species richness was highest in the Tropical Pacific and Tropical Indian Oceans, despite these regions exhibiting low TD (Figure 6). This is due to the high number of sea snakes in the region (Sikdar et al. 2025). Thus, similarly to previous studies, we found that areas with high species richness do not necessarily have high taxonomic distinction (Ellingsen et al. 2005), preventing these measurements from being surrogates of each other. Data is lacking when it comes to marine reptile distributions. However, these results are consistent with the theory of greater marine species richness in the Indo‐Pacific (Miller et al. 2018). These findings could be influenced by past biogeographic phenomena.

An example is the distribution of sea snakes, a group that has solely been observed in the Indian and Pacific Oceans, with the open water sea snake, the Yellow‐bellied Sea Snake *Hydrophis platurus* , which can be found in the Oriental Pacific (Lillywhite et al. 2014) being the only exception. Current distributions of sea snakes were caused by the barrier created by the Isthmus of Panama, which was formed before the arrival of the Yellow‐bellied Sea Snake and impeded its migration to the Atlantic (Lillywhite et al. 2018). Additionally, the lower temperatures in the South Atlantic along the coasts of Southern Africa act as additional barriers that prevent the migration of sea snakes towards the Atlantic (Lillywhite et al. 2018). However, increasing oceanic temperatures could create favorable conditions in this area that would enable future migration of sea snakes towards the Atlantic. Thus, marine reptile distributions could change substantially in response to climate change‐induced temperature changes.

### 4.3 Conservation Implications

Species from diverse taxa follow core‐to‐transition distributions worldwide, dispersing from areas of high endemicity, species richness, and taxonomic dissimilarity to surrounding transitional areas (Bernardo‐Madrid et al. 2025). Core areas can thus be identified through species richness and taxonomic distinctness, with higher taxonomic distinctness suggesting a higher necessity for the conservation of that region (Gallão and Bichuette 2015; Mondaca‐Fernández et al. 2017; Salvo Tierra et al. 2021; Bernardo‐Madrid et al. 2025). Accordingly, we identify key conservation hotspots based on our analyses. Our results document the phylogeographic differences between marine reptiles and mammals and their subgroups. For example, our results further support a decline of marine reptiles since the Mesozoic era and the established marine mammal lineages, which were increasing up until recently (Benson et al. 2010; Steeman et al. 2009; Motani et al. 2017). However, anthropogenic pressure has affected marine mammals and reptiles alike, with threats from habitat loss, contamination, overfishing, and climate change (Ceballos and Ehrlich 2002; Witt et al. 2010; Plön et al. 2024; Torres‐Romero et al. 2024, 2025). Thus, both groups should be equally protected (Dinerstein et al. 2019).

Marine Protected Areas (MPAs) have to be expanded to ensure the efficient protection of marine biodiversity (Rodríguez‐Rodríguez and Martínez‐Vega 2022). Currently, 8% of the oceans are protected, with only 3% in a highly protected zone. Following the United Nations Conference on Biological Diversity (COP15) held in Canada, the goal of protecting 30% of the oceans by the year 2030 was established. This study raises questions on whether MPAs could be used to protect lineage diversity in addition to areas of high biodiversity. These protected areas should focus on the Indo‐Pacific region for the conservation of marine reptiles, especially sea snakes. Additionally, MPAs should be focused on the Arctic and North Pacific regions for marine mammal lineage conservation. Arctic mammals have shown the highest levels of habitat loss and range reduction due to their sensitivity to increasing temperatures (van Weelden et al. 2021). In addition, protecting the high seas has been supported by previous research (Roberts et al. 2025).

Marine species face several threats, including bycatch, climate change, and habitat loss among others. Because of this, several methods have been developed for the management of their populations (Rasmussen et al. 2011; Báez et al. 2024). For sea turtles, Wallace et al. (2010) proposed the use of *Regional Management Units* (RMUs) to define both the threat distributions and population units. For marine mammals, *Important Marine Mammal Areas* (IMMAs) were created for the identification of crucial zones that can be delineated and managed in future conservation plans (Tetley et al. 2022). As of now, assessments for IMMAs in the Arctic Ocean have been fragmented (Starkweather et al. 2021; Marine Mammal Protected Areas Task Force 2025).

This study did not incorporate environmental factors in each region. Future research should continue assessing these distributions in relation to environmental conditions. Additionally, future studies could identify key conservation regions within the zones we found with high taxonomic distinction (e.g., through abundance of individuals per species, specific breeding or foraging grounds etc.; similar to: Hamann et al. 2010; Wallace et al. 2010; Wallace et al. 2011; Collen et al. 2014; Rodrigues et al. 2014; Colston et al. 2020; Ennen et al. 2021; Ennen et al. 2020). Another limitation of this study is the large sizes of the regions tested. Despite the capability of taxonomic distinctness to compare regions of distinct sizes, conservation efforts require smaller zones. Thus, future studies could assess specific distributions in the regions we found to be of importance in this study.

## Author Contributions

**Laura Fuentes‐Tejada:** formal analysis (equal), resources (equal), software (equal), writing – original draft (equal). **Davinia Torreblanca:** investigation (equal), resources (equal), software (equal), writing – review and editing (equal). **José Carlos Báez:** conceptualization (lead), funding acquisition (lead), methodology (lead), writing – review and editing (equal).

## Funding

This study was financially supported 374 by the project “Plan Complementario de I+D+i en el área de Biodiversidad (PCBIO)” funded by the 375 European Union within the framework of the Recovery, Transformation and Resilience Plan – 376 NextGenerationEU, by the Spanish Ministry of Science, Innovation and Universities and by the 377 Regional Government of Andalucia.

## Conflicts of Interest

The authors declare no conflicts of interest.

## Supporting information


**Data S1:** ece372537‐sup‐0001‐DataS1.xlsx.

## Data Availability

The data used is included in the Supporting Information.
